# Boosting Ionic Conductivity by Ordering Nanoparticles within All-Polymer Poly(ethylene oxide) (PEO) Nanocomposites

**DOI:** 10.1021/acspolymersau.5c00077

**Published:** 2025-09-10

**Authors:** Jorge L. Olmedo-Martínez, Gabriele Lingua, Leire Unanue, Monika Król, Janne Ruokolainen, Alejandro J. Müller, David Mecerreyes

**Affiliations:** † POLYMAT University of the Basque Country UPV/EHU, Avenida Tolosa 72, 20018, Donostia-San Sebastián, Spain; ‡ Department of Applied Physics, School of Science, Aalto University, FIN-00076 Espoo, Finland; § POLYMAT and Department of Polymers and Advanced Materials: Physics, Chemistry and Technology, Faculty of Chemistry, 160665 University of the Basque Country UPV/EHU, Paseo Manuel de Lardizabal, 3, 20018 Donostia-San Sebastián, Spain; ∥ IKERBASQUE, Basque Foundation for Science, Plaza Euskadi 5, 48009 Bilbao, Spain

**Keywords:** Ordering nanoparticles, single-ion conduction, Li-nanoparticles, ionic conductivity, poly(ethylene oxide), crystallization

## Abstract

In this work, we demonstrate that the ordering of ion-conducting nanoparticles within the interlamellar regions of semicrystalline poly­(ethylene oxide) (PEO) enhances its ionic conductivity. Specifically, lithium sulfonamide functional polymeric methacrylic nanoparticles (NPs) measuring 26.4 ± 5.6 nm were aligned within a PEO matrix by controlling the crystallization rate of PEO. Polarized light optical microscopy (PLOM) revealed that the temperature range between 52 and 56 °C allows for sufficiently slow crystallization kinetics to achieve NP ordering. This ordering was observed by using transmission electron microscopy (TEM) and small-angle X-ray scattering (SAXS). The alignment of the NPs results in an 8-fold increase in the ionic conductivity of the nanocomposite polymer electrolyte at room temperature, exhibiting lithium single-ion conducting behavior and achieving a value of 4.6 × 10^–5^ S cm^–1^ at 80 °C.

## Graphical abstract

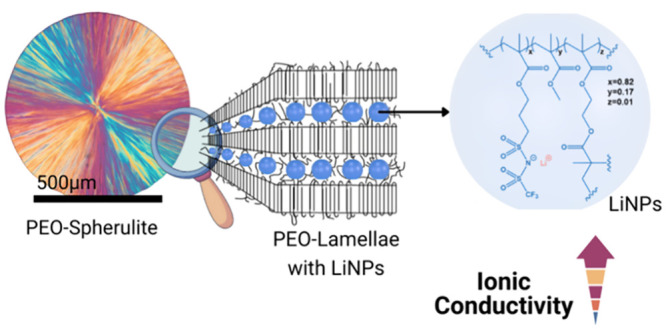

A popular method for enhancing the functional and mechanical properties of polymers is the preparation of nanocomposites by adding inorganic nanoparticles, carbon nanotubes, or cellulose nanofibers. The ordering or alignment of the nanocharges in the polymeric matrices can further improve certain properties of the nanocomposites, such as electrical conductivity and mechanical strength. This alignment of the nanoparticles/nanofibers within the polymer matrix can be achieved through different techniques, i.e., through block copolymer assembly, electrospinning,^1^ or applying different stimuli such as an electric or a magnetic field.^2–5^ Interestingly, Kumar et al. recently showed that crystallization at very low supercoolings can also be employed to order inorganic NPs, such as surface-functionalized SiO_2_, into the interlamellar amorphous regions of the spherulites formed by PEO, by controlling the crystallization rate.^6,7^ Namely, when the spherulite growth rate is below a critical value, the NPs can order into the amorphous interlamellar regions of the semicrystalline spherulitic superstructure, improving their mechanical properties.^8^

Inorganic nanoparticles (NPs) are commonly added to poly­(ethylene oxide) (PEO) to form “disordered” nanocomposite electrolytes used in lithium solid-state batteries to enhance both their mechanical and ion transport properties.^9^ Some of us have recently shown that polymer NPs can be used to prepare all-polymer nanocomposites to obtain lightweight PEO polymer electrolytes.^10^ Interestingly, it was also shown that functionalizing the surface of the polymer nanoparticle with lithium sulfonamide groups gives the possibility of obtaining polymer electrolytes with lithium single-ion conducting behavior.^11–13^ It is worth mentioning that lithium single-ion conducting electrolytes are actively sought since they bring advantages such as lithium dendrite suppression to lithium metal battery technology.^14–19^

In this letter, we show that the ordering of polymeric NPs within the interlamellar regions of PEO can be used as an efficient tool to improve the ionic conductivity of nanocomposite polymer electrolytes (Figure 1a). For this purpose, we first investigated the crystallization kinetics of the nanocomposite to determine the optimal conditions for NP ordering. The PEO/NP nanocomposites were based on lithium sulfonamide functional polymeric nanoparticles of poly­(MMA-*co*-LiMTFSI), named LiNP, synthesized by emulsion polymerization, with an average diameter of 26.4 ± 5.6 nm, as previously reported (DLS presented in Figure S1),^12^ and *T*_*g*_ of 110 °C.^13^ A solvent casting method was used to prepare the all-polymer nanocomposites. The spherulitic growth rate was measured between 52 and 57 °C for nanocomposites of PEO, including 15 wt % LiNPs (Figure 1b-1c). This is important for determining the temperature range in which PEO crystallizes slowly enough to allow NP ordering.

**Figure 1:**
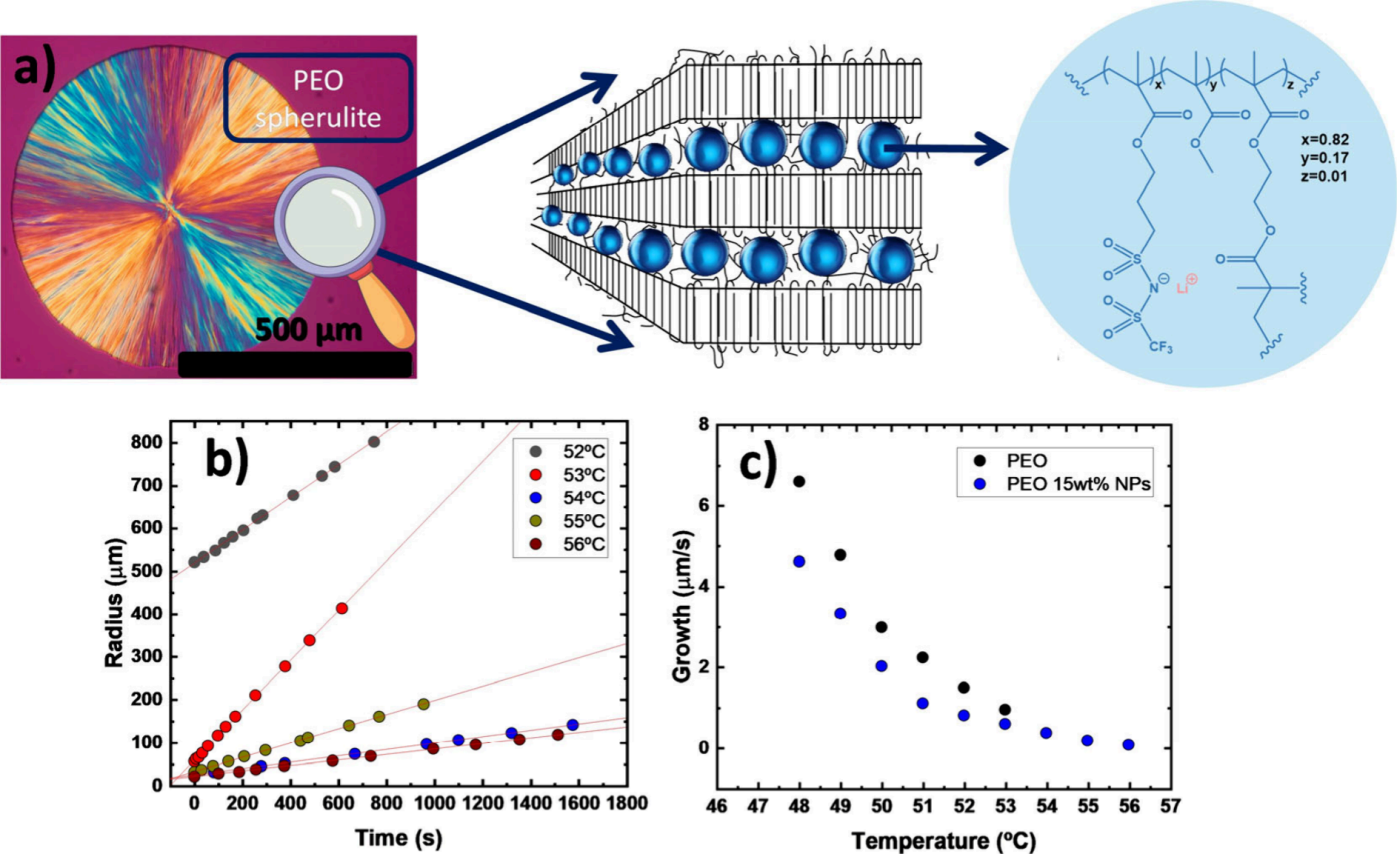
a) Representation of the arrangement of NPs in between PEO crystalline lamellae, b) PEO spherulite radius as a function of time at different temperatures for PEO 15 wt % NPs, and c) Spherulitic growth rate as a function of time for neat PEO and PEO 15 wt % NPs.

The growing crystalline lamellae affect the dispersion of NPs based on the relative ratio of the crystal growth rate to the NP diffusivity.^20^ This interplay is the basis for NP ordering. Specifically, the crystalline front pushes the moving NPs into the amorphous interlamellar regions^21^ for sufficiently slow spherulitic growth rates. Figure 1b presents the radius of the studied spherulite at each temperature as a function of time. The slope is less steep as the temperature increases, indicating that the growth rate decreases. In addition, it is important to verify that the growth of the spherulites is constant as a function of time. Figure 1c shows the spherulitic growth rate as a function of temperature for neat PEO and PEO with 15 wt % LiNPs. It is observed that the crystallization rate of PEO decreases when mixed with the NPs, indicating that the NPs interact with the polymer, reducing the crystallizability of PEO.^22^ Also, in the case of the nanocomposite of 15 wt % NP PEO, the crystallization rate decreases with increasing temperature up to 56 °C. An attempt was made to calculate the spherulitic growth rate at 57 °C. However, after 24 h, no spherulites were observed. A temperature between 52 and 56 °C seems ideal for slow spherulite growth. The methodology used to prepare the samples consists of increasing the temperature to erase the thermal history of the material, then cooling rapidly at 60 °C min^–1^ to the selected crystallization temperature, then leaving it at this temperature for 48 h for the PEO to crystallize slowly and finally lowering the temperature to 25 °C. It is important to mention that the temperature accuracy in the DSC and in the Linkam chamber is ±0.1 °C. After this thermal treatment, the ordering of the NP within the PEO was investigated by Small Angle X-ray Scattering (SAXS) and Transmission Electron Microscopy (TEM). First, SAXS was measured at 100 °C in the molten state, and also at room temperature on nonisothermally crystallized nanocomposites from the melt (disordered) and after isothermal crystallization at *T*_*c*_ = 52 °C (ordered). Figure 2a presents the Lorentz corrected intensity as a function of the scattering vector (*q*) for polymer nanocomposites with different thermal treatments. It is observed that the sample in the molten state at 100 °C is characterized by the absence of any SAXS signal, neither from PEO nor from the NPs. This indicates that when the polymer is molten, the cross-linked NPs do not have enough contrast to be detected by SAXS. When cooling from the molten state at 20 °C min^–1^, the material crystallizes nonisothermally during cooling, and at 25 °C, the sample exhibits a SAXS reflection at 0.33 nm^–1^. On the other hand, when the sample that was isothermally crystallized at 52 °C is measured at 25 °C, a reflection at a smaller *q* value (0.25 nm^–1^) is observed. We interpret these maxima as the long period of the crystalline lamellae within the PEO spherulites, assuming that the included NPs do not produce any SAXS scattering. Therefore, the average long period (*d* value) (calculated *d* = 2*πn* **/** *q* ^*^) is increasing from 19.2 to 24 nm following isothermal crystallization at 52 °C. Interestingly, this peak shift to a lower *q* number probably indicates that the long period (*d*) between the PEO lamellae^23^ increases due to the presence of the NPs within the interlamellar amorphous polymer. The discrepancy in the NPs size measurements obtained via DLS and SAXS arises from the fundamental differences between the two techniques. In DLS, the presence of the dispersion medium (water) may induce a slight swelling of the NPs, potentially leading to larger apparent sizes. In contrast, SAXS provides an average value of all long periods present in the sample, reflecting the structural periodicity rather than the hydrodynamic diameter.

**Figure 2:**
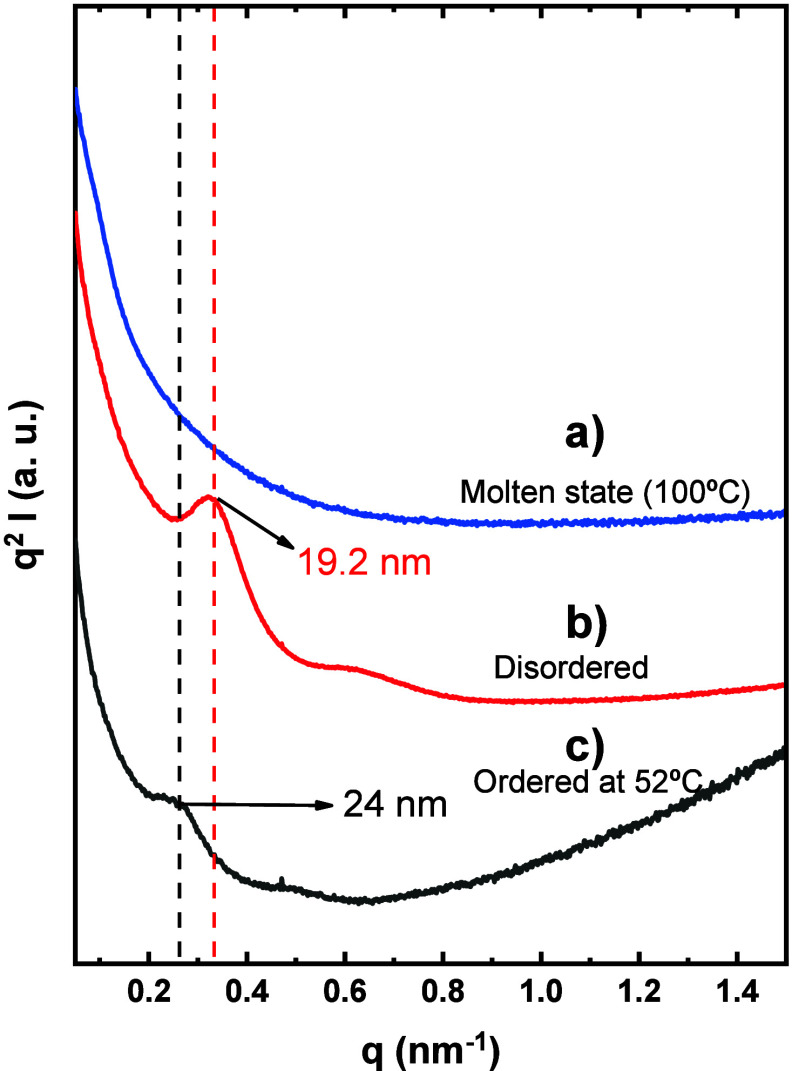
Lorentz-corrected SAXS of blend PEO 15 wt % NPs at a) Molten state (100 °C), b) Crystallized nonisothermally from the melt at 20 °C min^–1^ (disordered) and, c) Ordered at 52 °C.

Another way to visualize the ordering of the NPs in the PEO matrix is through TEM. Figure S2 presents TEM images of the disorder sample, where good NP dispersion in the PEO matrix is observed. Figure 3 presents two different zones of the ordering of the NPs after PEO isothermal crystallization at 52 °C, and Figure 3a clearly shows the lamellar morphology. Figure 3b offers a magnified view of the nanoparticles (NPs), enabling estimation of their average size, which is approximately 25 nm. Previous reports in the literature showed aligned NPs within PEO spherulites that employed inorganic nanoparticles (bare or functionalized silica), and in that case, the samples observed by TEM were not stained. In those previous cases, the electron density contrast between PEO and silica was very high, so the silica NPs could be observed by TEM or cryo-TEM with a high resolution.^24^ In our case, the nanocomposite samples had to be stained with RuO_4_ to allow the visualization of the organic NPs which have very low electron density contrast, similar to PEO. After staining, the organic nanoparticles and the amorphous part of PEO are observed in a dark gray color, while the crystalline part of PEO is light gray. PEO crystallization at 52 °C provoked the alignment of the lithium sulfonamide functional polymer nanoparticles, as seen in the micrographs presented in Figure 3, even if the resolution is not as good as when inorganic silica NPs are employed. In the SI, Figures S2–S3 show the TEM at different crystallization temperatures, where some ordering is also detected, although 52 °C seems to be the ideal isothermal crystallization temperature for ordering the NPs within the interlamellar amorphous regions.

**Figure 3:**
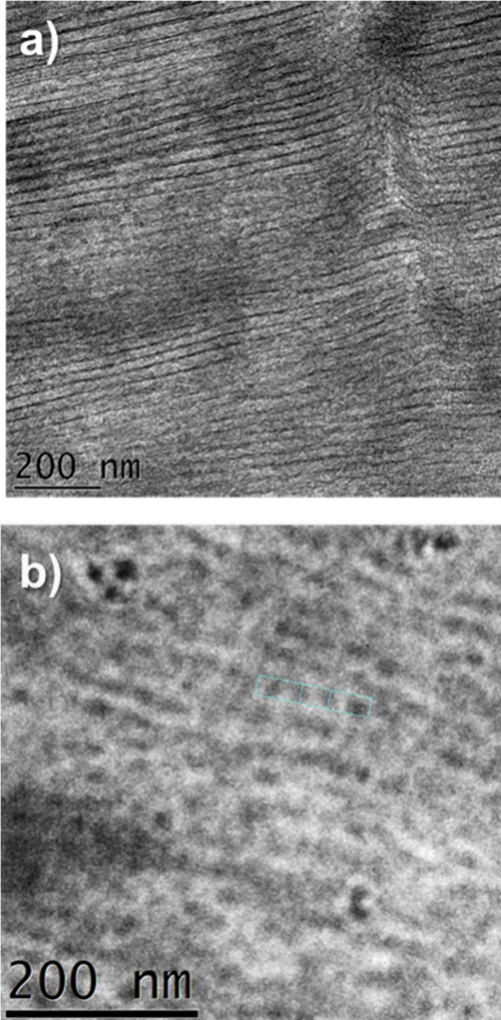
TEM micrographs of a PEO nanocomposite including 15 wt % of polymer NPs ordered by isothermal crystallization at 52 °C.

Finally, we investigated the effect of ordering the NPs on the ionic conductivity of the PEO/NPs all-polymer nanocomposite electrolytes. Electrochemical impedance spectroscopy (EIS) with 20 mV amplitude between the 300 kHz and 0.1 Hz frequency range was used to extrapolate the impedance values. The Nyquist plots of EIS for all samples at different temperatures are illustrated in Figure S4. In the case of the disordered sample, the electrolyte was cooled from the melt at 20 °C min^–1^ to 25 °C. Samples from the same batch were used for all experiments to have comparable impedance values.

Each sample was placed in a coin cell between two stainless steel electrodes with a silicon separator (6 mm Ø and 500 μm thickness), and then the cell was heated at 80 °C for 5 h to erase the thermal history of the sample before being treated at different isothermal crystallization temperatures, *T*_*c*_, for 48 h. Then, the cell was cooled down to 25 °C before performing the ionic conductivity test versus temperature to mimic the ordering method elucidated before. The test was performed between 25 and 80 °C with a 10 °C step, with an equilibration time of 1 h at each temperature.

As displayed in Figure 4a, the absolute impedance value (|Z|) of the disordered sample differs from those of the ordered ones. We observed that samples treated at 55 and 52 °C present lower |Z| values in the whole range of temperatures. Namely, the sample treated at 52 °C showed the lowest |Z| values and, consequently, the highest ionic conductivity (Figure 4b). Meanwhile, a further decrease in the isothermal crystallization temperature to 45 °C leads to a higher impedance and lower ionic conductivity. The |Z| values of the sample crystallized at 45 °C are similar or even lower than those of the disordered sample (crystallized nonisothermally at 20 °C/min), indicating that the exact crystallization temperature for slow spherulite growth is fundamental to achieve the optimal NP ordering along with the highest ionic conductivity. Notably, the final limited time at 80 °C during the electrochemical test is insufficient to promote NP disorder, and the impedance at 25 °C after the temperature ramp does not significantly change. This behavior can be explained by considering different scenarios of nanoparticle ordering as a function of the crystallization temperature. For sufficiently slow spherulitic growth rates, the polymer crystalline front pushes the mobile nanoparticles, which are hierarchically ordered in the amorphous interlamellar, interfibrillar, and interspherulitic regions (of the spherulitic semicrystalline morphology). Thus, depending on the crystallization rate, three types of NP distribution can be obtained,^8^ and this distribution can be related to the ionic conductivity as follows: (i) NPs are engulfed within the spherulites at random locations and NPs are spatially well dispersed (this occurs at very fast crystallization, which presents the lowest ionic conductivity), (ii) NPs are assembled in sheets within the interlamellar regions of the spherulites (slow crystallization, which presents the highest values of ionic conductivity), and (iii) NPs are pushed by the growth front toward interfibrillar and/or interspherulitic regions (very slow crystallization, which again lowers the ionic conductivity of the electrolyte). This would indicate that when trying to order the NPs at crystallization temperatures lower than 52 °C we would be in the first case, in the range between 52 and 55 °C we would be in the second case, explaining that it would be the optimal range to increase the ionic conductivity, and temperatures higher than 55 °C would have NPs pushed toward interfibrillar/interspherulitic zones, which causes a decrease in the ionic conductivity.

**Figure 4:**
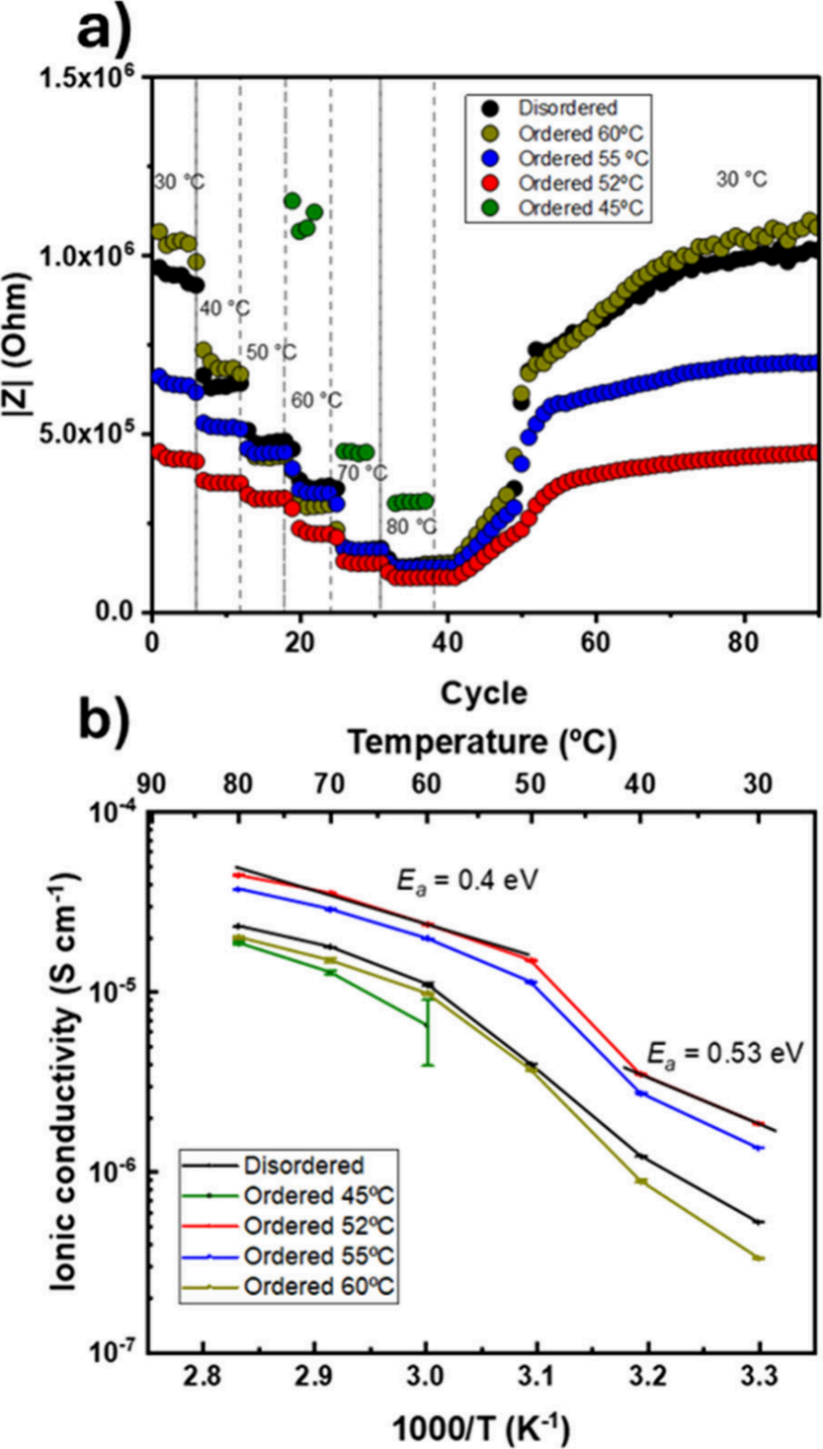
a) Absolute impedance variation over cycles at different temperatures and b) ionic conductivity vs temperature of all polymer electrolytes with *E*_*a*_ of sample “Ordered at 52 °C”. The error bar corresponds to the standard deviation from the average log­(σ) obtained by measuring four values at each temperature.

The ionic conductivity vs temperature plot for all samples was extrapolated to corroborate the results by fitting the Nyquist plots reported in Figure S4 with the equivalent circuit illustrated in Figure S5. As already observed in the |Z| vs cycle plot, the sample ordered at 52 °C displayed the highest ionic conductivity in the whole range of temperature, reaching 2.0 × 10^–6^ and 4.6 × 10^–5^ S cm^–1^ at 30 and 80 °C, respectively. Despite the partial melting of the PEO at around 50 °C, it seems the effect of the nanoparticle ordering in the polymer electrolytes is retained, and the difference in ionic conductivity is clearly detectable even at 80 °C in the fully amorphous state of PEO. Notably, the ionic conductivity values of the sample ordered at 52 °C at 30 °C are equal or higher than those of several single-ion conducting polymers reported in the literature, considering that the highest ionic conductivity values in the interval between 80 and 100 °C are in the range of 10^–4^ S cm^–1^.^25^ Indeed, the typical drop in conductivity associated with the crystallization of PEO around 60 °C is less noticeable due to the addition of nanoparticles, which act as plasticizers, reducing the crystallinity of the polymer scaffold, as already reported in our previous work. Table S1 shows the spherulitic growth rates for the samples ordered at different temperatures, as well as the corresponding ionic conductivity values.

Aiming to understand if the ordering of the single-ion NPs affects the transport of Li^+^ ions through the polymer electrolyte, the lithium-ion transfer number (*t*_*Li*_^*+*^) of samples crystallized at 52, 55, and 60 °C was calculated. The measurement was performed at 50 °C to eliminate the possible influence of unwanted thermal treatment at higher temperatures, which could affect NP ordering. Indeed, because of the general high resistances of the electrolytes at 52 °C, the resulting *t*_*Li*_^*+*^ values were calculated both following the Bruce-Vincent method and through the ratio of the initial current to the steady-state current using the chronoamperometry plot reported in Figure S6.^26^ The *t*_*Li*_^*+*^ values evaluated with different methods are reported in Table S2. The sample treated at 52 °C presents the highest value of 0.67, while samples crystallized at 55 and 60 °C show similar values of 0.54 and 0.57, respectively. These results confirm that the ordered all-polymer composites show similar lithium single-ion conducting behavior than the nonordered ones while showing superior ionic conductivity.

In conclusion, this work demonstrates that the alignment of nanoparticles within semicrystalline PEO boosts its ionic conductivity. Thus, lithium sulfonamide functional polymeric methacrylic nanoparticles (NPs) were aligned within the poly­(ethylene oxide) (PEO) matrix by controlling the crystallization rate of PEO. It was found that between 52 and 56 °C is the temperature range in which the crystallization kinetics is sufficiently slow to achieve the ordering of the NPs in the interlamellar regions of the spherulites. This ordering was observed by transmission electron microscopy (TEM) and small-angle X-ray scattering (SAXS). The distribution of the NPs was also related to the ionic conductivity obtained, finding that when PEO crystallizes isothermally between 52 and 55 °C NPs assembled in sheets, which increases the ionic conductivity of the electrolyte. The alignment of the NPs provokes an increase in the ionic conductivity of the nanocomposite polymer electrolyte by eight times at room temperature while showing a similar lithium single-ion conducting behavior.

## Supplementary Material



## Notes

In the Supporting Information there are the materials used in the development of the work, the experimental techniques, as well as the way of obtaining the PEO 15 wt % NPs blend, in addition to the DLS of the NPs in water (Figure S1). TEM images at different crystallization temperatures (Figure S2 and S3). The Nyquist plots as a function of temperature (Figure S4), the equivalent circuit used (Figure S5), the chronoamperometry results with transport number table (Figure S6 and Table S2) for the electrolytes at different ordering temperatures, and Table S1 with the Spherulitic growth rate and ionic conductivity values for all the samples ordered at different crystallization temperatures (PDF)

## #

Jorge L. Olmedo Martínez – Universidade da Coruña, Campus Industrial de Ferrol, CITENI, 15403, Ferrol, Esteiro, Spain
