# Supplementary material for: Boosting Ionic Conductivity by Ordering Nanoparticles within All-Polymer Poly(ethylene oxide) (PEO) Nanocomposites
Source: ACS Polym Au. 2025 Sep 10;5(5):488–93. doi: 10.1021/acspolymersau.5c00077 (PMC12511981; doi:10.1021/acspolymersau.5c00077)
Supplement: Supplementary file 1 [file lg5c00077_si_001.pdf]

Supplementary information

Boosting Ionic Conductivity by ordering nanoparticles within all-polymer PEO Nanocomposites

Jorge L. Olmedo-Martínez [a#*], Gabriele Lingua [a], Leire Unanue [a], Monika Król [b], Janne Ruokolainen [b], Alejandro J. Müller [c,d*], David Mecerreyes [a,d*]

[a] POLYMAT University of the Basque Country UPV/EHU, Avenida Tolosa 72, 20018, Donostia-San Sebastián, Spain.

[b] Department of Applied Physics, School of Science, Aalto University, FIN-00076 Espoo, Finland

c POLYMAT and Department of Polymers and Advanced Materials: Physics, Chemistry and Technology, Faculty of Chemistry, University of the Basque Country UPV/EHU, Paseo Manuel de Lardizabal, 3, 20018 Donostia-San Sebastián, Spain.

[c] IKERBASQUE, Basque Foundation for Science, Plaza Euskadi 5, 48009, Spain

Present Addresses

[#] Universidade da Coruña, Campus Industrial de Ferrol, CITENI, 15403, Ferrol, Esteiro, Spain

*Emails: jorge.olmedo.martinez@gmail.com, alejandrojesus.muller@ehu.es, david.mecerreyes@ehu.eus

Materials

Poly(ethylene oxide) (100 000 g mol^{-1}, Sigma-Aldrich), methyl methacrylate (MMA, Sigma-Aldrich), ethylene glycol dimethacrylate (EGDMA, Sigma-Aldrich), and lithium 1-(3-(methacryloyloxy)propylsulfonyl)-1-(trifluoromethylsulfonyl)imide (LiMTFSI) were purchased from Specific Polymers. Lithium dodecyl sulfonate (LiDS, Sigma-Aldrich), ascorbic acid, *tert*-butyl hydroperoxide (70% solution in water, TBHP), and acetonitrile (ACN, Sigma-Aldrich).

Preparation of the electrolyte

LiNPs were synthesized using the methodology proposal by Gallastegui et al [3]. In this work blends of PEO 15wt% LiNPs were prepared because it was necessary to have a material with high crystallinity (as we reported in our previous work [4]). The all-polymer nanocomposite was prepared by a solvent casting method; PEO (1 g) was completely dissolved in acetonitrile (ACN) (20 mL), after which the polymer nanoparticles were added (176 mg), and the solution was ultrasonicated for 30 min. The solvent was evaporated at room temperature for 24 h; then, the sample was placed in a vacuum oven at 70 °C to remove all remaining solvent.

Experimental Techniques

PLOM

To know the range of temperatures where PEO crystallized slow in the sample PEO 15wt% LiNPS. The spherulitic growth rate was measured using an OLYMPUS BX51 polarized light microscope fitted with an OLYMPUS SC50 camera and a Mettler FP82HT hot stage with liquid nitrogen cooling capability. The samples were placed between two glass slides and heated 20 °C above their melting temperature (using the DSC measurements as the reference) to form a thin film and kept at this temperature for 3 min to erase thermal history. The samples were then cooled at 50 °C/min to a temperature at which the spherulites began to appear, and the growth of the spherulite was followed isothermally as a function of time by recording micrographs.

DSC

To order the LiNPs in PEO matrix, different temperatures were evaluated (between 50-60 °C). These experiments were carried out using a PerkinElmer 8000 DSC instrument with an intracooler II, calibrated with tin and indium as standards. The mass for each evaluated temperature was around 50 mg. The sample was heated from 25°C to 100°C at 20 °C/min and held at 100°C for 10 min to erase the thermal history and to be completely sure that all the polymer is melted, then cooled to the selected T_c at 60 °C/min and holding at this temperature for 24 h. Finally decrease the temperature to 25°C at 20 °C/min.

SAXS experiments

SAXS experiments were performed at room temperature after thermal treatment. These experiments were performed at the BL11-NCD beamline in the ALBA Synchrotron in Barcelona (Cerdanyola del Vallés, Spain). To carry out the heat treatment, the samples were placed in aluminum pans (the same ones used in the DSC). SAXS was measured on the nanocomposites without thermal annealing treatment (disordered) and with thermal annealing process (order). Interestingly, a peak shift to lower q number was observed, indicating that the long period (d) between the PEO lamellae (equation S1) increases due to the presence of the NPs within the interlamellar amorphous polymer.

$$d = \frac{2\pi n}{q^*} \tag{S1}$$

DLS of neat LiNPs

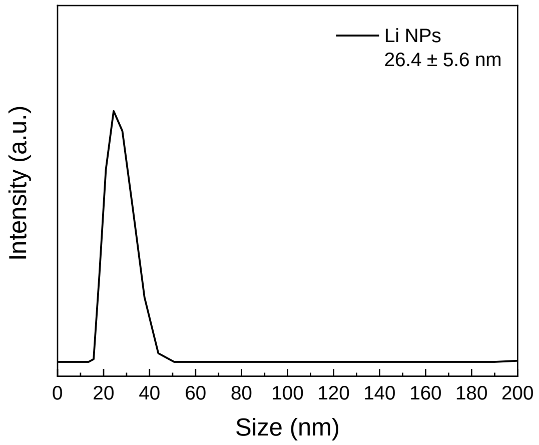

Figure S1. DSL of NPs

Transmission Electron Microscopy (TEM)

Polymer sections, 70 nm thick, were obtained using a Leica EM UC7 ultramicrotome at −160 °C with a 35° Diatome diamond knife. The resulting sections were deposited onto 300-mesh copper grids coated with lacey carbon. The grids were subsequently vacuum-dried and restored to room temperature. Following this, the sections were stained by exposure to RuO_4 vapor with varying times (1 s and 10 min) prior to imaging. It was observed that with the shorter staining time, the structures of NPs were more visible, whereas with the longer, PEO features.

Cryo-transmission electron microscopy (cryo-TEM) was performed using a JEOL JEM-3200FSC field-emission microscope operated at an accelerating voltage of 300 keV. The instrument was equipped with an omega-type zero-loss energy filter, with a slit width of 30 keV. The bright-field micrographs were recorded using a Gatan Ultrascan 4000 CCD camera. Throughout data acquisition, the specimen temperature was maintained at −187 °.

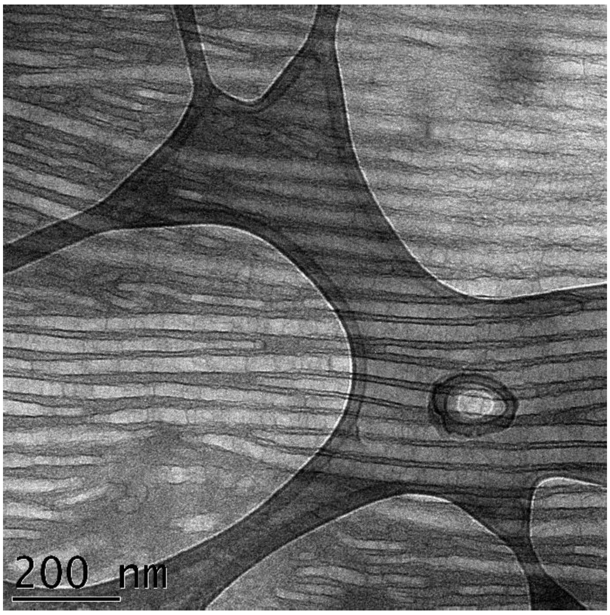

Figure S2. PEO 15wt% NPs ordered at 54ºC

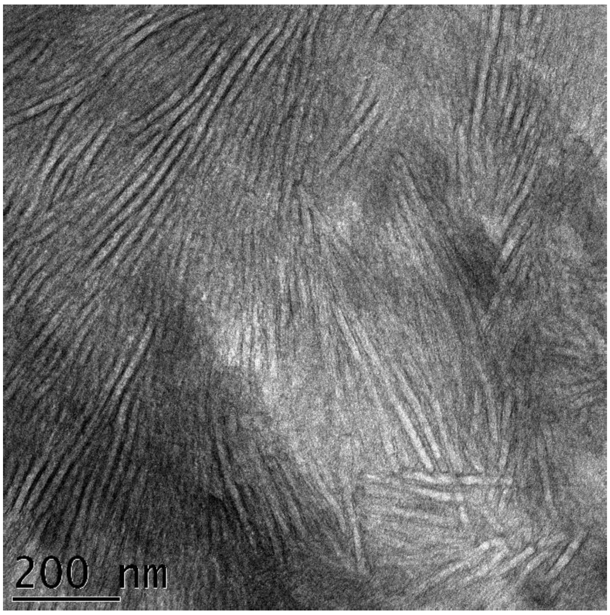

Figure S3. PEO 15wt% NPs ordered at 56ºC

Ionic conductivity

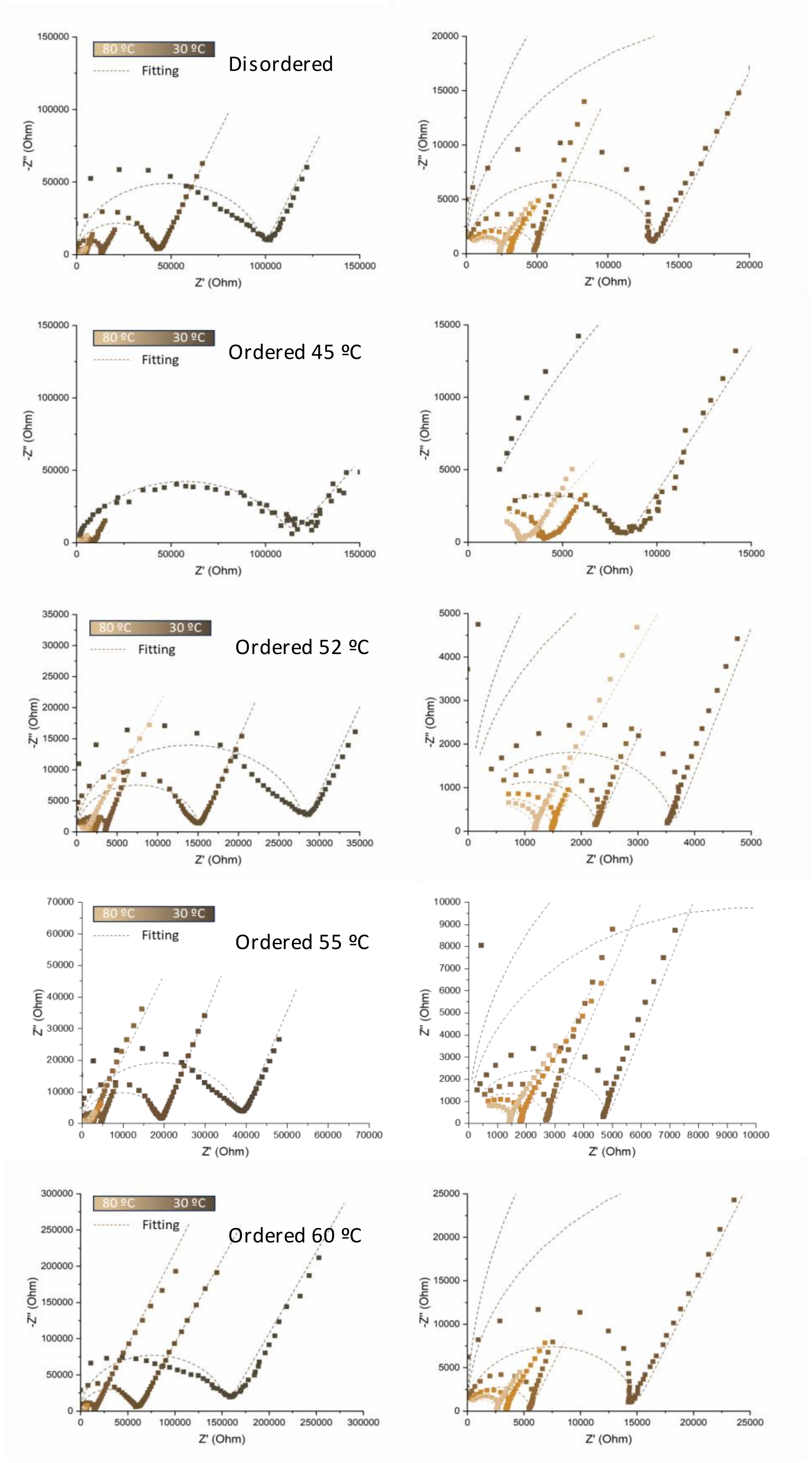

Figure S4. Nyquist plot vs temperatures of PEO/NP SICPEs treated at different temperatures in the full scale (left). Zoom of the graphs are displayed on the right.

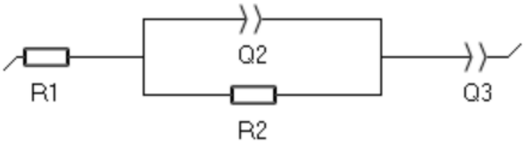

Figure S5. Equivalent circuit used to adjust the Nyquist plots.

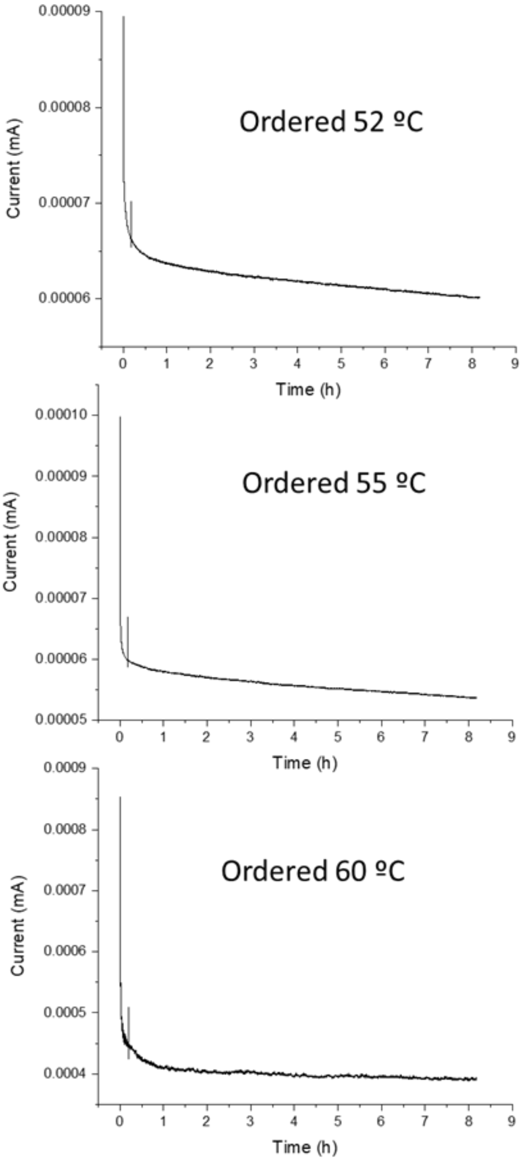

Figure S6. Chronoamperometry results of different ordered PEO/NP samples.

Table S1. Spherulitic growth rate and ionic conductivity values for all the samples ordered at different crystallization temperatures.

	Spherulitic growth rate	Ionic conductivity with SD error (S cm^{-1})		
**Sample**	**Microns/s**	**30 ºC**	**60 ºC**	**80 ºC**
Ordered at 45 ºC	5.14	-	6.81E-06 ± 2.60E-06	1.89E-05 ± 2.60E-06
Ordered at 52 ºC	0.8	2.01E-06 ± 9.59E-09	2.45E-05 ± 2.95E-07	4.58E-05 ± 4.39E-07
Ordered at 55 ºC	0.16	1.38E-06 ± 3.70E-09	1.80E-05 ± 6.10E-08	3.71E-05 ± 4.19E-08
Ordered at 60 ºC	-	3.40E-07 ± 3.15E-09	9.62E-06 ± 1.11E-07	2.01E-05 ± 2.86E-07
Disordered		5.31E-07 ± 1.66E-09	1.17E-05 ± 1.66E-09	2.31E-05 ± 1.15E-07

Table S2. Data obtained from chronoamperometry and Nyquist plot to calculate the t_{Li^+} values with different methods as reported in equation *a* and *b*.

Sample	T (°C)	Time	Area (cm^2)	Current (I, mA)	R_b (Ohm)	ΔV (V)	Iss *Rb,ss (mA Ohm)	I0*Rb,0 (mA Ohm)	t_+ [a]	$t_+(I_{ss}/I_0)$ [b]
Ordered at 52 ºC	50	t_0	1.54	8.90E-08	1505000	0.1	9.19E-02	1.34E-01	**0.69**	**0.67**
	50	t_{ss}	1.54	6.00E-08	1531000					
Ordered at 55 ºC	50	t_0	1.54	9.98E-08	1151000	0.1	6.54E-02	1.15E-01	**0.57**	**0.54**
	50	t_{ss}	1.54	5.36E-08	1221000					
Ordered at 60 ºC	50	t_0	1.54	6.88E-07	343108	0.1	1.36E-01	2.36E-01	**0.58**	**0.57**
	50	t_{ss}	1.54	3.92E-07	346756					

a) $t_{Li^+} = \frac{I_{ss}\, R_{b,ss}\,(\Delta V)}{I_0\, R_{b,0}\,(\Delta V)}$

b) $t_{Li^+} = \frac{I_{ss}}{I_0}$
